# Evaluation of Scheimpflug Tomography Parameters in Subclinical Keratoconus, Clinical Keratoconus and Normal Caucasian Eyes

**DOI:** 10.4274/tjo.89587

**Published:** 2018-06-28

**Authors:** Samira Huseynli, Farah Abdulaliyeva

**Affiliations:** 1 National Ophthalmology Center Named After Academician Zarifa Aliyeva, Ophthalmology Clinic, Baku, Azerbaijan

**Keywords:** Subclinical keratoconus, keratoconus, Scheimpflug tomography, Pentacam

## Abstract

**Objectives:**

To evaluate tomographic and topographic parameters in subclinical and clinical keratoconus eyes by comparing them with normal eyes in a young Caucasian population.

**Materials and Methods:**

This cross-sectional study included 88 normal eyes (control group), bilateral data from the preclinical stage of 24 progressive keratoconus eyes (bilateral subclinical keratoconus group), 40 fellow eyes of patients with unilateral keratoconus (fellow eyes group) and 97 eyes with mild keratoconus (clinical keratoconus group). Topographic and tomographic data, data from enhanced elevation maps and keratoconus indices were measured in all study eyes using Scheimpflug tomography. Receiver operating characteristic (ROC) curve analysis was used to assess individual parameters to discriminate eyes of patients with subclinical and clinical keratoconus from control eyes. The sensitivity and specificity of the main effective parameters were evaluated and optimal cut-off points were identified to differentiate subclinical keratoconus and keratoconus from normal corneas.

**Results:**

Comparison of all subclinical and clinical keratoconus eyes from the normal group revealed significant differences in most diagnostic parameters. The ROC curve analysis showed high overall predictive accuracy of several Pentacam parameters (overall D value, anterior and posterior elevations and difference elevations, pachymetry progression index, index of surface variance, index of height decentration and keratoconus index) in discriminating ectatic corneas from normal ones. These outcomes were proportionally less pronounced in all subclinical keratoconus eyes than in the clinical keratoconus eyes. Pachymetric readings were progressively lower in the bilateral subclinical keratoconus eyes and sensitivity and specificity of the analyzed tomographic and topographic parameters were higher than the fellow eyes group when differentiating subclinical keratoconus from healthy corneas.

**Conclusion:**

Scheimpflug tomography parameters such as D value, elevation parameters, progression index and several surface indices can effectively differentiate keratoconus from normal corneas in a Caucasian population. Nevertheless, a combination of different data is required to distinguish subclinical keratoconus.

## Introduction

Keratoconus (KC) is a corneal ectatic disorder, usually bilateral in most cases, characterized by progressive corneal thinning resulting in corneal protrusion, irregular astigmatism and decreased vision.^1,2^ Modern advances in computer-based technologies and imaging techniques have increased our ability to diagnose KC. Thus, determining the incidence of subclinical KC (ScKC) and clinical KC will provide a more accurate estimation of the impact of such new treatment options on healthcare costs.^3^ The incidence of KC varies depending on factors such as ethnicity and the criteria used to establish the diagnosis; most estimates place the incidence in the general population between 50 and 230 per 100,000, though rates vary greatly in different geographic regions.^4^ Screening for clinical KC is not difficult due to its corneal topography and biomicroscopic, retinoscopic and pachymetric findings. However, detection of this ectatic disorder is difficult at the very early or preclinical stages. The identification of corneas at higher risk or susceptibility represents a major challenge for refractive surgeons.^5^

Early detection of KC is closely related to the clinical care of these patients. These patients should not be assigned to refractive laser treatment but rather should undergo further screening for an ectatic disorder to detect progressive ectasia. Abnormal preoperative topography and age were reported to be the most significant predictive variables for ectasia development.^6^

The term ScKC describes the very early preclinical stage of KC that can only be detected with diagnostic examinations such as corneal topography. Much effort has been made to implement these data for patient screening in refractive surgery and several different approaches have been attempted to discriminate a cornea with ScKC and a normal cornea using corneal topography.^7^ However, exact diagnosis of ScKC is still difficult, as there is a lack of defined threshold criteria. A major reason for that difficulty is that persons with suspected bilateral KC continue in their suspected status until definitive KC develops in one eye. Nevertheless, due to lack of symptoms in the early stages, patients often present with advanced KC. Studies revealed differences in the corneal topographic pattern between normal eyes and eyes with presumed ScKC, as represented by fellow eyes or eyes of family members of KC patient, or eyes that developed postLASIK ectasia.^8,9,10^

The Scheimpflug camera we used is considered to be the most sensitive device to detect early forms of KC. It uses various indices derived from tomographic thickness evaluation parameters, such as the corneal thickness spatial profile, the percentage of thickness increase and Belin/Ambrosio Enhanced Ectasia Display (BAD). BAD utilizes both anterior and posterior elevation data and pachymetric data to screen for ectatic change.^11,12,13^ The purpose of this study was to analyze the keratometric, topometric and pachymetric properties of early keratoconic corneas of Caucasian eyes with the Scheimpflug imaging camera and to study the usefulness of different indices in differentiating ScKC and clinical KC eyes from normal eyes.

## Materials and Methods

In this cross-sectional study, we evaluated patients who visited the clinic and underwent Pentacam HR examination. The local ethics committee of the Zarifa Aliyeva National Ophthalmology Center approved the study and it was conducted according to the principles set forth in the Declaration of Helsinki. Prior to examination, every participant gave his/her informed consent and the patient anonymity was preserved. Inclusion criteria were minimum age of 17 years and definitive findings consistent with KC, such as those described by the Collaborative Longitudinal Evaluation of Keratoconus group.^14^ ScKC was diagnosed using criteria defined in previous studies,^15,16,17,18,19,20,21,22,23^ including corneal topography with abnormal localized steepening or an asymmetric bow-tie pattern, a normal-appearing cornea on slit-lamp biomicroscopy and at least 1 of the following signs: steep keratometric curvature (>47.0 overall deviation [D]), oblique cylinder >1.5 D, central corneal thickness less than 500 mm and being the fellow eye of clinical KC, with or without abnormal topography. According to the Scheimpflug KC indices, ScKC eyes were categorized as being normal, with a Pentacam KC system indication of 0.

Control cases were selected from a database of candidates for refractive surgery with normal corneas and myopia or myopic astigmatism. Eyes were considered normal if they had no ocular pathology, no previous ocular surgery and no irregular corneal pattern on corneal tomography. One eye was randomly selected from each candidate for inclusion in this study. Exclusion criteria included a history of corneal surgery, significant corneal scarring and significant ophthalmic disease that might potentially affect the outcomes.

In the study we used the WaveLight Oculyzer II (Alcon Surgical, Ft Worth, Texas), a Pentacam High-Resolution Scheimpflug imaging camera 26 (Oculus Optikgeräte GmbH, Wetzlar, Germany), running on software version 1.17r47. The readings were taken as recommended in the instruction manual of the instrument.^24^ Image quality was checked and for each eye only one examination with a high quality factor was recorded. Various parameters were derived from topographic and topometric maps and the BAD as described below.

Data obtained from topographic maps: mean keratometric readings along the flattest (K1) and steepest (K2) meridians, topographic astigmatism (cylinder) and asphericity for the anterior corneal surfaces, maximum curvature power on front of the cornea with vertical, horizontal location absolute distance from apex in mm, corneal thickness at the center (central corneal thickness) and at the thinnest point of the cornea (thinnest corneal thickness). The absolute distances from the corneal apex to the thinnest point of the cornea were determined. 

Data obtained from the BAD: Corneal height data measurement was followed by evaluation of elevation of the thinnest point from 8 mm anterior and posterior, by using a conventional best-fit sphere (BFS) as the reference surface (in mm) and corneal elevation difference values were taken as the differential changes in corneal elevation between the BFS and the enhanced BFS (with exclusion of a 3.5-mm optical zone in the thinnest portion of the cornea).

The BAD also contains five new terms (D values for standard deviation [SD] from the mean) representing the front surface, back surface, pachymetric progression, thinnest point and thinnest point displacement. The D is the final overall map reading taking each of the five parameters into account. Each individual parameter D and the final D reported as SDs from the mean were also recorded. Progression index is calculated as the average progression value at different pachymetric rings, referenced to the mean curve. The average, minimum and maximum pachymetric progression indexes were recorded. 

Corneal volume (CV) is reported as the volume of the cornea in a diameter of 3, 5 and 7 mm, centered on the anterior corneal apex. 

Data obtained from topometric maps: Corneal parameters such as index of surface variance, index of vertical asymmetry, keratoconus index (KI), central keratoconus index, index of height asymmetry and index of height decentration were evaluated as additional tools in differentiating KC from healthy eyes with thin corneas.

### Statistical Analysis

Statistical analysis was performed using SPSS software (version 23.0; SPSS Inc., Chicago, IL, USA). ANOVA was used to test differences for age among the groups. Considering all indices in the KC group were non-normally distributed, the analyzed parameters were compared among the groups using the non-parametric Kruskal-Wallis test, post hoc analysis was done with Mann-Whitney U test with Bonferroni correction to compare each pair of groups. The results are expressed as mean ± SD and a value of p<0.05 was considered statistically significant. Receiver operating characteristic (ROC) curves were used to determine the overall predictive accuracy of the parameters when used as a test to identify eyes with KC. The diagnostic specificity and sensitivity of the 10 most effective parameters were evaluated and compared with ROC and cut-off points were presented. 

## Results

Ninety-seven eyes of 97 patients (80 males/17 females) with mild KC (KC group, Pentacam system indication TKC 1), 88 eyes of 64 patients (60 males/4 females) with ScKC (ScKC group; Pentacam system indication TKC 0) and 88 eyes of 88 candidates for refractive surgery (55 males/33 females) with normal corneas (normal group) were analyzed. Mean age was 22.19±2.97, 21.5±3.13 and 21.5±2.95 years respectively in the KC, ScKC and normal groups. Among the ScKC patients, 24 eyes of 12 patients were included in the bilateral ScKC subgroup and 40 eyes in the unilateral ScKC subgroup. Preclinical stage data of both eyes in patients with documented progressive KC were included in the bilateral ScKC group. All eyes in the bilateral ScKC group had suspicious tomography and topography findings and a 1- to 3-year follow-up period showed KC progression in 1 or both eyes. Patients who were diagnosed with clinical KC in 1 eye and had no slit-lamp findings and no topography finding significant enough to be diagnosed as clinical KC in the fellow eye were included in unilateral ScKC subgroup. The mean Pentacam parameters and the differences between clinical and ScKC patients are shown in Tables 1 and 2.

We found no significant differences in terms of mean and maximum keratometry or astigmatism between the ScKC and control eyes (p≥0.07, Kruskal-Wallis test). However, all other values were significantly different between the analyzed groups (Table 2).

Comparison of bilateral ScKC eyes to the fellow eyes of clinical KC eyes revealed significant differences in corneal thickness variables (CCT, ThCT) (p<0.01, Mann-Whitney U test). The CV (CV 3-7) values showed lower distribution in the bilateral ScKC group than in the unilateral KC group (p<0.01, Kruskal-Wallis test). However, other diagnostic variables showed no significant differences between the groups.

Pairwise comparisons among the clinical KC and other groups of eyes revealed the following significant differences: keratoconic versus normal eyes, all variables (p<0.01, Mann-Whitney U test); keratoconic versus fellow eyes, all variables except Thin L.Dist Abs, CV7; and KC versus bilateral ScKC eyes, all variables except flat keratometry, astigmatism and volume values.

### Receiver Operating Characteristic Curve Analysis

When discriminating fellow eyes with ScKC from control eyes, the D value showed the highest AUC (0.904), followed by posterior elevation (0.887) (Table 3).

In discriminating between bilateral ScKC eyes and control eyes, most parameters had high AUCs (Table 3); however, corneal thickness and volume parameters showed higher AUCs than in other groups.

Between the clinical KC and normal groups, the diagnostic efficiency of most characteristic parameters increased significantly (all AUC>0.9), indicating their excellent discrimination capacity. However, posterior elevation at the thinnest point, the overall D value and KI showed the highest AUCs (Table 3).

Table 4 shows the cut-off points and sensitivity and specificity values of the main effective Pentacam parameters derived from ROC curve analysis in all study groups.

**Figure 1:**
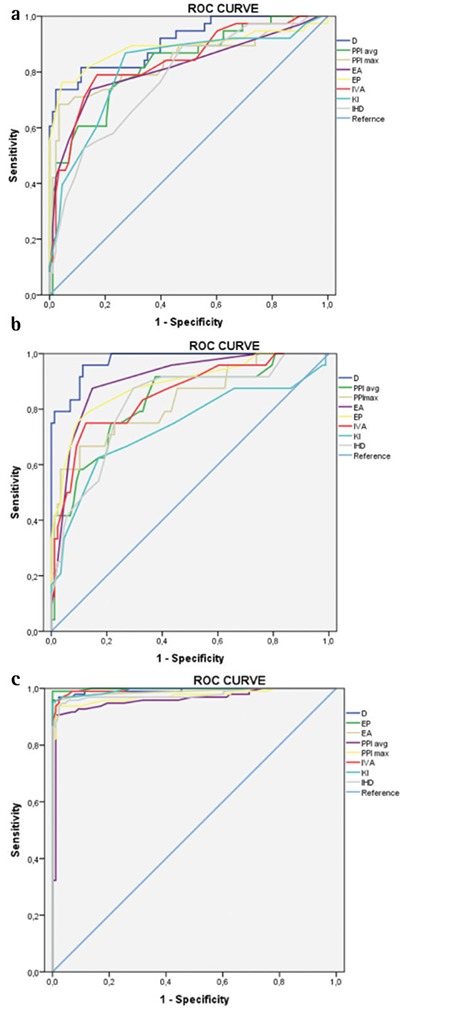
Receiver operating characteristic curves of main effective Pentacam parameters to detect unilateral subclinical keratoconus (a), bilateral subclinical keratoconus (b) and clinical keratoconus (c) D: Overall deviation, EA: Anterior elevation at the thinnest point, EP: Posterior elevation at the thinnest point, PPI: Pachymetric progression index, IVA: Index of vertical asymmetry, KI: Keratoconus index, IHD: Index of height decentration

Figure 1 presents graphical representations of the ROC curves of main effective Pentacam parameters with higher predictive accuracy to detect subclinical and clinical KC. 

## Discussion

The pathogenesis of primary KC remains unclear. As known from the literature, KC is generally a bilateral disorder, although initially only one eye might be affected. We also know that approximately 50% of the unaffected fellow eyes will progress to KC within 16 years. In a study by Li et al.^9^ more than one-third of clinically normal eyes in patients with unilateral KC developed manifest KC during the 8-year follow-up period. Several studies investigated early screening and diagnosis of KC using the Pentacam device in different ethnic populations.^16,17,18,19,20,21,22,25,26,27,28,29,30,31^ Results varied in different populations related to race, geographic location and size of the study population (Table 5).

Most such studies differ from each other by the criteria used to diagnose subclinical/forme fruste KC.^16,17,18,19,20,21^

The aim of the present study was to identify and compare characteristics of the subtle morphologic changes in bilateral KC-suspect eyes and clinically normal fellow eyes of patients with KC. In our study, all subclinical eyes had no clinical signs of KC but had abnormal topographic features with asymmetric bowtie and focal or inferior steepening pattern. According to the Scheimpflug camera, KC indices of these eyes were categorized as being normal (with system indication “0”). Thus, analysis of these eyes might help to identify at-risk corneas, especially in refractive surgery candidates. 

In this study, D value was the most characteristic index between all analyzed groups and showed the highest area under the ROC curve, followed by posterior and anterior elevation. We found that the best cut-off for D value to differentiate clinical KC from controls was 1.83 with 100% sensitivity and 96.0% specificity. On the other hand, the best cut-off for D value in differentiating eyes with bilateral ScKC from normal eyes was 1.73 with a sensitivity of 96.7% and specificity of 79%, suggesting excellent sensitivity and specificity. However, when differentiating fellow eyes of unilateral KC eyes from normal eyes, the best cut-off for D value was 1.59 with excellent sensitivity (95.5%) but limited specificity (73.7%).

The D value is a multimetric combination parameter composed of keratometric, pachymetric, pachymetric progression and posterior elevation parameters. Muftuoglu et al.^18^ showed that among the keratometric, pachymetric (including progression indices) and posterior elevation indices, D value had the best areas under the ROC curve to differentiate between clinical and ScKC eyes and control eyes. They found that the best cut-off for D value to differentiate KC from controls was 2.1, with 100% sensitivity and 100% specificity. This result suggests that the new D index can be valuable as a sole parameter in diagnosing KC. But the best cut-off for the D value in differentiating eyes with ScKC from normal eyes was 1.3, with 60% sensitivity and 90% specificity, suggesting good specificity to diagnose ScKC but limited sensitivity. 

In another study population, the Pentacam’s suspicious cut-off for overall D value was >1.61 as optimal for their particular keratoconic sample.^20^ Considering a suspicious D value (>1.6 SD) as positive in order to maximize sensitivity while sacrificing specificity, they preferred to falsely flag a cornea as ectatic than to miss a ScKC case during the preoperative evaluation of refractive surgery candidates. 

In our study, the D value was significantly different in the KC, ScKC and healthy groups; these results are very comparable to those of other studies.^18,20,21^ However, our study included only patients diagnosed with mild KC.

Posterior elevation was the most discriminating parameter between eyes with ScKC and controls in our study, consistent with a report by de Sanctis et al.^25^ In their study, posterior elevation showed high predictive accuracy for ScKC compared to the controls (AUC=0.93) and the optimal cut-off was 29 µm, with 68% sensitivity and 90.8% specificity. Due to differencesin acquiring points of the device, Du et al.^26^ reported a much smaller cut-off value for posterior elevation (7.5 µm) but with a comparable sensitivity (70.7%) and specificity (93.8%). In our study, anterior and posterior elevations in the analyzed study groups were significantly different; however, as displayed in Table 5, we obtained much lower values than those reported in other studies, especially in the KC group. This may be explained by the use of newer software and the fact that we utilized elevation indices at the thinnest point from 8 mm BFS. Uçakhan et al.^17^ evaluated Pentacam parameters in ScKC compared with normal eyes. They defined ScKC as the fellow eye of KC and found that corneal thickness distribution indices and posterior elevation are more helpful than anterior curvature data in identifying eyes with ScKC. Additionally, they also evaluated the anterior/posterior elevation depression difference and suggested that posterior elevation difference was the strongest discriminating factor, followed by anterior elevation depression. The anterior and posterior elevation difference values were available in the BAD display software for the Pentacam proposed by Villavicencio et al.^13^ Anterior and posterior corneal elevation differences determined with enhanced BFS may provide more accurate diagnostic information for KC than the amounts of anterior and posterior corneal elevation themselves determined with conventional BFS.^17^^,18,19,25,26,27,28,29,30^

Kamiya et al.^30^ observed in Japanese patients that anterior and posterior elevation measurements tended to have a higher accuracy at the earlier stages of KC, so they concluded that elevation and elevation difference measurements might provide useful information to improve the diagnostic accuracy in early KC. They detected that posterior elevation (0.980) and anterior elevation (0.977) showed the highest areas under the ROC curve. Their results are highly comparable to ours in AUROC of indices.

Pinero et al.^16^ reported progressively lower pachymetric readings in eyes with subclinical, early, or moderate KC (p<0.01).The CV was significantly lower in the moderate KC group than in the subclinical and mild groups. A possible explanation for this finding may be that at early stages of KC a redistribution of CV occurs with no loss of tissue. As discussed, we found significant differences in CCT, ThCT, CV3, CV5 and CV7 between normal eyes and eyes with subclinical or clinical KC. 

Additionally, in our study the bilateral ScKC group showed lower distribution in corneal thickness parameters and CV (CV 3-7) values than fellow eyes of the clinical KC eyes and these parameters had higher predictive accuracy than when comparing the fellow eye group to normal eyes. An explanation of this finding could be that subclinical eyes with low pachymetric reading showed a greater tendency toward progression. Using the Pentacam, Bae et al.^19^ evaluated topographic and tomographic changes in fellow eyes of Asian patients with unilateral KC to compare them with normal eyes. Previous research indicates that true unilateral KC is very rare, thus the normal fellow eye may be the ideal model for the mildest form of ScKC. The group found that fellow eyes in unilateral KC patients showed differences in several parameters that were not detectable with the Pentacam detection program. In their study on ROC curve analysis, keratometric asymmetry and topometric index were best at discriminating fellow eyes from normal, followed by elevation differences on the posterior and anterior corneal surface. In our study from anterior surface Pentacam-derived topometric indices, the index of surface variance, index of height decentration and KI were the most sensitive and specific criteria to diagnose ScKC. This is comparable to some previous studies.^21,22^

In this study we found significantly increased topographic elevation, pachymetry and topometric values in bilateral suspect eyes and fellow eyes of patients with unilateral KC compared with the values in control eyes. We also found that corneal topography and tomography outcomes were proportionally less pronounced in all ScKC eyes than in clinical KC eyes. Comparing bilateral suspect eyes from fellow eyes of patients with unilateral KC, we found that eyes in the former subgroup have more cornea tissue alteration than the latter subgroup. Furthermore, sensitivity and specificity of the analyzed tomographic and topographic parameters were significantly higher in the former subgroup than the latter group compared to the values in control eyes. 

This study has some limitations, including a higher proportion of males than females in the study group. The preponderance towards males in the population is consistent with the authors’ clinical experience of the male/female incidence in keratoconic patients and KC incidence studies and thus, this is unlikely to skew the results of this study.^22,31^

## Conclusion

In conclusion, this study showed that several Petacam parameters, such as BAD D value, anterior and posterior elevation and difference elevation, pachymetry progression index, index of surface variance, index of height decentration and KI are very effective in discriminating KC from normal corneas. The current study supports findings previously reported on the usefulness of Scheimpflug imaging to assess subclinical keratoconic eyes in different population and confirm results indicating that any single parameter taken alone is not sufficient to distinguish normal cornea from one with ScKC, as the studied parameters showed some degree of overlap in normal and pathologic corneas. Further studies with a larger number of patients and with controls composed of a relevant clinical population and simultaneous evaluation of the corneal biomechanics and wavefront aberrations would be useful to diagnose early KC in the Caucasian population. 

## Figures and Tables

**Table 1:**
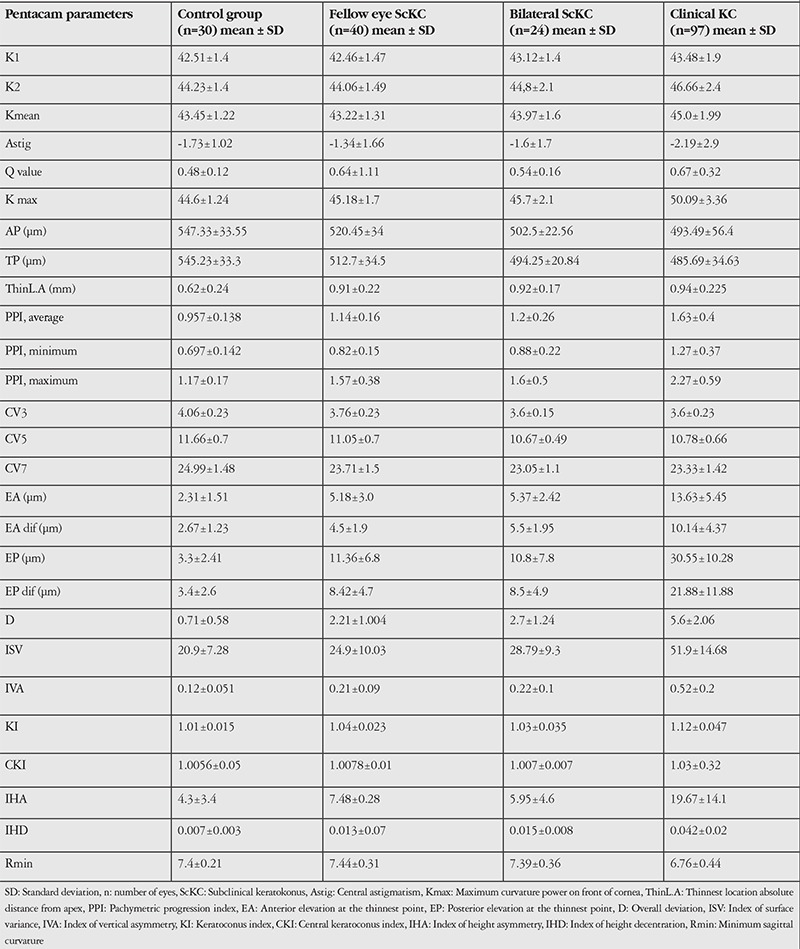
Mean Pentacam parameters between subclinical, clinical keratoconus and normal eyes

**Table 2:**
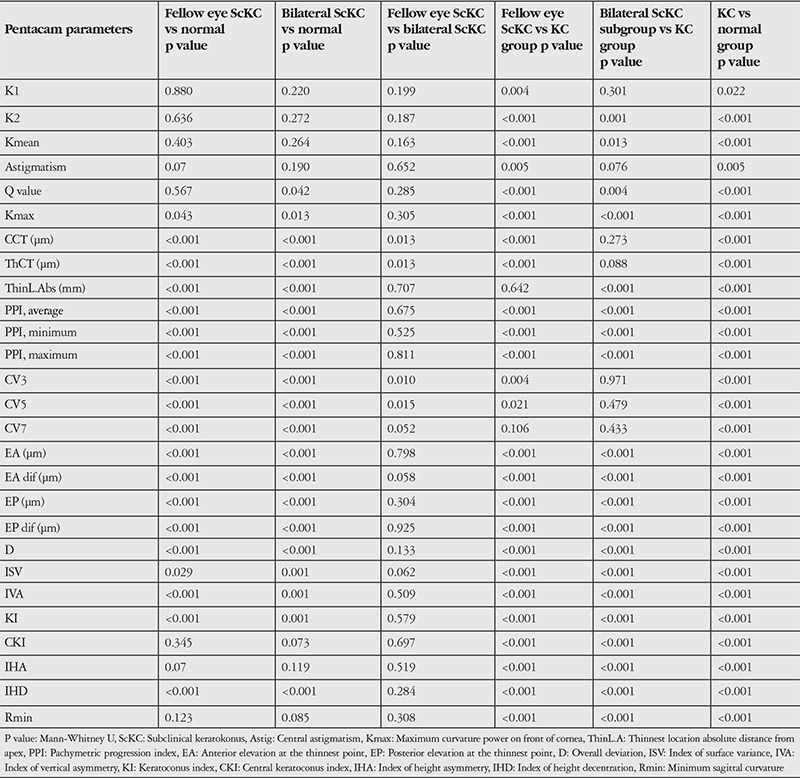
Comparison of Pentacam parameters between normal, bilateral subclinical keratokonus, fellow eye of the unilateral keratokonus and clinical keratoconus eyes

**Table 3:**
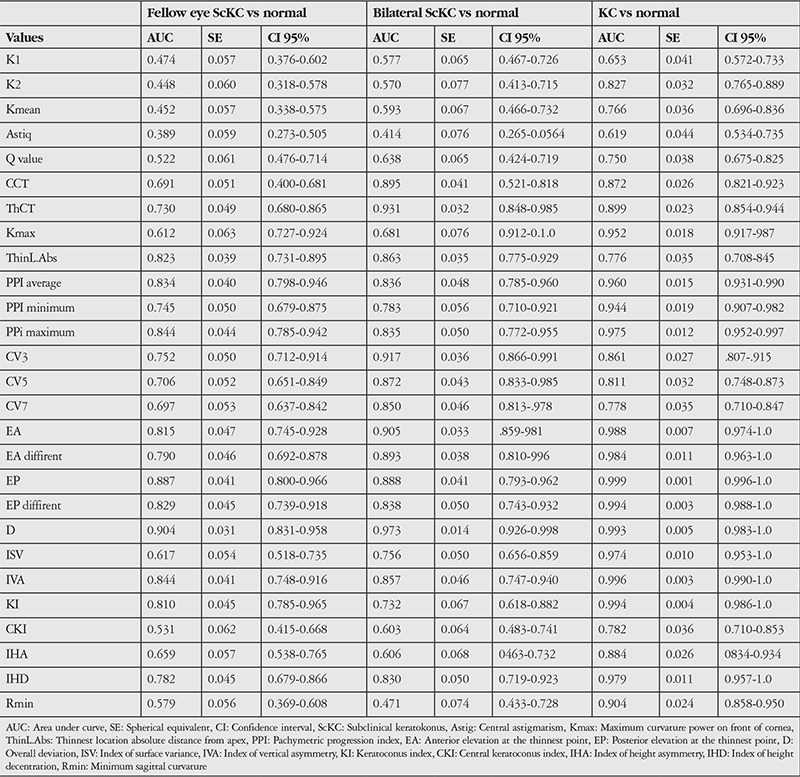
Receiver operating characteristic curve analysis for subclinical and clinical keratoconus eyes versus normal eyes

**Table 4:**
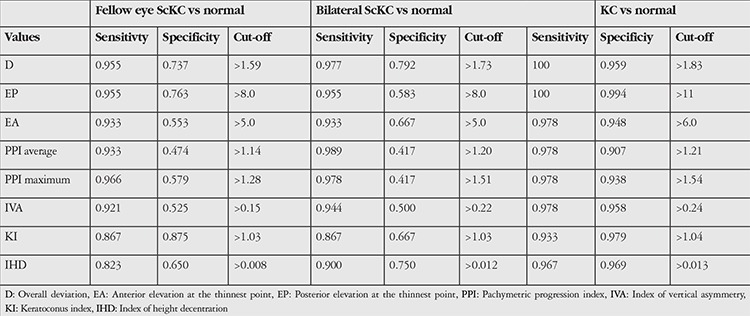
Cut-off points, sensitivity and specificity of the main effective Pentacam parameters derived from receiver operating characteristic curve analysis

**Table 5:**
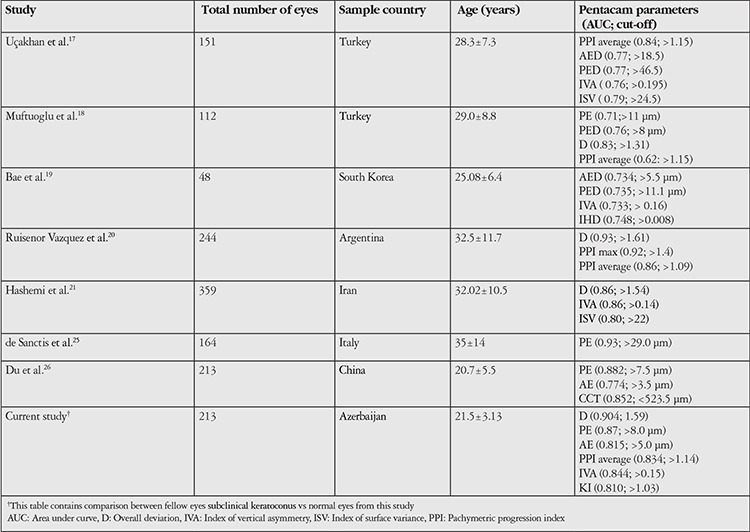
Summary of the studies of effective Pentacam parameters to detect subclinical keratoconus eyes
